
JOURNAL INFORMATION
==============================
NLM Title Abbreviation: Access Microbiol
Journal ID: Access Microbiol
NLM Journal ID: 101746927
Journal ID: acmi

Access Microbiology

EISSN: 2516-8290
Publisher: Microbiology Society

ARTICLE INFORMATION
==============================
PMCID: PMC7472549
Article version: 1
Subjects: Poster Presentation

Abstracts from the British Yeast Group Meeting 2019

Publication date: 2019
Electronic publication date: 2019 Nov 29
Volume: 1
Issue: 9
Electronic Location ID: 4
Copyright: © 2019 The Authors
Copyright year: 2019
License: This is an open-access article distributed under the terms of the Creative Commons Attribution License.
License URL: https://creativecommons.org/licenses/by/4.0/

==============================


Assessing the ecological role of yeasts in the human gut

http://jmmcr.microbiologyresearch.org

Ward Grace *
Gibson Glenn
Wijeyesekera Anisha
Sanderson Jeremy
Brostoff Jonathan
The University of Reading , Reading , United Kingdom
Guy’s and St Thomas’ NHS Foundation Trust , London , United Kingdom
*Correspondence:Grace Ward, grace.ward@pgr.reading.ac.uk

Comparative Genomics of the Saccharomyces complex

http://jmmcr.microbiologyresearch.org

Keane Ann-Marie *
Elliston Adam
Roberts Ian
Dicks Jo
Quadram Institute , Norwich , United Kingdom
Earlham Institute , Norwich , United Kingdom
*Correspondence:Ann-Marie Keane, ann-marie.keane@quadram.ac.uk

Hog1 signalling in the emerging multidrug resistant fungal pathogen Candida auris

http://jmmcr.microbiologyresearch.org

Bamborough Leon *
Quinn Janet
Day Alison
Newcastle University , Newcastle , United Kingdom
*Correspondence:Leon Bamborough, l.bamborough2@newcastle.ac.uk

Evaluating the use of variation graphs for the characterisation of yeast rDNA arrays

http://jmmcr.microbiologyresearch.org

Ursani Ziauddin *
Davey Robert
Dicks Jo
Quadram Institute Bioscience , Norwich , United Kingdom
Earlham Institute , Norwich , United Kingdom
*Correspondence:Ziauddin Ursani, ziaursani@yahoo.com

Development of a new high-throughput method for screening large yeast libraries for use in the beverage industry

http://jmmcr.microbiologyresearch.org

Aguiar-Cervera Jose *
Severn Oliver
Singer Instruments , Watchet , United Kingdom
The University of Manchester , Manchester , United Kingdom
*Correspondence:Jose Aguiar-Cervera, jose@singerinstruments.com

The activity of S. pombe LTR retrotransposons is activated in response to rapamycin

http://jmmcr.microbiologyresearch.org

Chan Tsun Ho
Whitehall Simon *
Newcastle University , Newcastle upon Tyne , United Kingdom
*Correspondence:Simon Whitehall, simon.whitehall@ncl.ac.uk

Dissecting the role of Peroxiredoxins in regulating conserved ROS-activated kinases

http://jmmcr.microbiologyresearch.org

Galler Martin *
Day Alison
Latimer Heather
Vos Harmjan
Quinn Janet
Dansen Tobias
Veal Elizabeth
Newcastle University , Newcastle upon Tyne , United Kingdom
University Medical Center Utrecht , Utrecht , Netherlands
*Correspondence:Martin Galler, m.galler2@newcastle.ac.uk

Inhibition of Drug-Resistant Candida glabrata by Killer Toxins Produced by Saccharomyces cerevisiae

http://jmmcr.microbiologyresearch.org

Fredericks Lance *
Roslund Cooper
Crabtree Angela
Rowley Paul
University of Idaho , Moscow , USA
*Correspondence:Lance Fredericks, fred6557@vandals.uidaho.edu

Drug resistant Candida colonization among ICU patients in a Nigerian teaching hospital

http://jmmcr.microbiologyresearch.org

Abiri Oyekola *
Obafemi Awolowo University Teaching Hospitals Complex , Ile-ife , Nigeria
*Correspondence:Oyekola Abiri, kolabiri@yahoo.com

Modification of Candida albicans cell wall by commensal gut bacteria

http://jmmcr.microbiologyresearch.org

Buzun Ekaterina *
Quinn Janet
Lowe Elisabeth
Newcastle University , Newcastle upon Tyne , United Kingdom
*Correspondence:Ekaterina Buzun, e.buzun@newcastle.ac.uk

